# Draft Genome Sequence of the Aureocin A53–Producing Strain *Staphylococcus aureus* A53

**DOI:** 10.1128/genomeA.00858-16

**Published:** 2016-08-25

**Authors:** Olinda Cabral Silva Santos, Andreza Freitas Souza Duarte, Rodolpho Mattos Albano, Maria Carmo Freire Bastos

**Affiliations:** a Departamento de Microbiologia Geral, Instituto de Microbiologia Paulo de Góes, Universidade Federal do Rio de Janeiro, Rio de Janeiro, Brasil; b Departamento de Bioquímica, Instituto de Biologia Roberto Alcântara Gomes, Universidade do Estado do Rio de Janeiro, Rio de Janeiro, Brasil

## Abstract

Here, we present the 2,658,363-bp draft genome sequence of the aureocin A53–producing strain *Staphylococcus aureus* A53. This genome information may contribute to the optimal and rational exploitation of aureocin A53 as an antimicrobial agent and to its production in large scale.

## GENOME ANNOUNCEMENT

*Staphylococcus aureus* A53 was isolated from pasteurized commercial milk (1) and produces an atypical class II bacteriocin, aureocin A53, a 51-residue peptide produced and secreted without any posttranslational modification (2). Aureocin A53 exhibits a broad spectrum of antimicrobial activity (3), being highly bacteriolytic against multidrug-resistant nosocomial staphylococcal strains (4), streptococci, and staphylococci involved in bovine mastitis (5, 6). It also exhibits an activity against strains of the foodborne pathogen *Listeria monocytogenes*, even in a food matrix, and several features relevant to application in food biopreservation (7). Therefore, aureocin A53 has a great potential for biotechnological applications.

Aureocin A53 is encoded by plasmid pRJ9 (1). The complete sequence of pRJ9 showed that this plasmid carries 14 open reading frames, from which eight are involved in aureocin A53 production and in immunity to this bacteriocin (2, 8). As bacteriocin synthesis is an energy-consuming process, it is generally closely controlled (9). However, as pRJ9 carries no functions involved in regulation of aureocin A53 production, these functions may be encoded on the bacterial chromosome. Description of the genome of strain A53 may help us in future investigations on the regulation of aureocin A53 production and, therefore, on how to increase aureocin A53 yields.

The sequencing library was prepared using the Nextera XT DNA sample preparation kit (Illumina) following the manufacturer’s recommendations. Whole-genome shotgun sequencing was performed on the Illumina MiSeq system. *De novo* assembly of 1,838,024 reads was conducted using the A5-miseq pipeline (10), yielding 142-fold average genome coverage and resulting in a draft genome comprising 27 scaffolds ranging from 16,057 to 277,790 bp that represent the single chromosome and the 10,406-bp plasmid pRJ9. Genome annotation was performed using the Rapid Annotation using Subsystem Technology (RAST) server (11) and by Prokka (12). The total scaffold size was determined to be 2,658,363 bp, featuring a G+C content of 32.7%. Gene prediction by Prokka revealed 2,418 coding sequences, 21 tRNA genes, and 5 rRNA genes.

Gene-category analysis showed that most genes are related to membrane transport and carbohydrate, lipid, and amino acid metabolisms. Genes involved in the regulation and expression of teicoplanin and fluoroquinolone resistances, as well as in the production of beta-lactamases and multidrug-resistance efflux pumps were also found. Moreover, genes encoding several virulence factors, including alpha-hemolysin, nonclassical enterotoxins, and proteins involved in biofilm formation, were also identified.

Availability of the *S. aureus* A53 draft genome sequence may contribute to the optimal and rational exploitation of aureocin A53 as an antimicrobial agent and to its production in large scale. The fact that the *S. aureus* A53 genome encodes secreted virulence factors also encourages the research of aureocin A53 production through heterologous expression of its gene cluster in staphylococcal species used in the food industry and graded as nonpathogenic (13).

### Accession number(s).

This whole-genome shotgun project has been deposited in DDBJ/ENA/GenBank under the accession number LVID00000000. The version described in this paper is the first version, LVID01000000. The plasmid pRJ9 sequence has been deposited at GenBank under the accession number AF4478813 (2).
